# Understanding the usefulness–risk paradox in AI writing tool adoption: An extended technology acceptance model study in Chinese University L2 writing contexts

**DOI:** 10.1371/journal.pone.0355494

**Published:** 2026-08-07

**Authors:** Tingxuan Li, Ziyu Liu, Deping Zou

**Affiliations:** Faculty of Foreign Languages, Qilu Normal University, Jinan, China; Public Library of Science, UNITED KINGDOM OF GREAT BRITAIN AND NORTHERN IRELAND

## Abstract

Generative artificial intelligence (GenAI) is rapidly reshaping higher education, and AI writing tools have become increasingly prominent in second language (L2) writing contexts. However, the mechanisms underlying students’ adoption of these tools, as well as the perceived consequences of their use, remain insufficiently understood. Grounded in an extended Technology Acceptance Model (TAM), this study examines how AI self-efficacy (ASE), multidimensional perceived risk, and behavioral engagement (BE) are associated with Chinese university students’ adoption of AI writing tools in L2 writing-related tasks and their perceived writing-related outcomes. A total of 518 valid questionnaires were collected from undergraduate students with prior experience using AI writing tools for English writing, revision, translation, or other L2 writing-related tasks, and structural equation modeling was employed to test the proposed acceptance–use–outcome framework. The results show that ASE was positively associated with perceived ease of use (PEOU) and perceived usefulness (PU). While PEOU was negatively associated with overall perceived risk (OPR), PU was positively associated with OPR, indicating that students who perceived AI writing tools as useful also tended to report stronger awareness of potential risks. OPR was not significantly associated with attitudes toward AI writing tools. Attitude was strongly associated with behavioral engagement, which in turn was positively associated with both perceived positive and perceived negative writing-related outcomes, with the positive association being substantially stronger. These findings extend TAM in AI-mediated learning by showing that functional value and risk awareness may coexist rather than offset one another in L2 writing contexts. Because the study used cross-sectional self-report data, the findings should be interpreted as associations among perceived constructs rather than evidence of direct causal effects or objective improvement in writing performance. The study also offers practical implications for the responsible integration of AI writing tools in higher education.

## 1. Introduction

### 1.1. Research background and problem statement

GenAI is rapidly reshaping teaching and learning in higher education. Among its emerging applications, AI writing tools such as ChatGPT have attracted particular attention because they can assist users with idea generation [1], language correction [2], text organization [3], and revision across multiple stages of the writing process [4]. In L2 writing contexts, these tools are often seen as especially promising, as they may help learners reduce linguistic barriers, improve writing efficiency, and access immediate support during drafting and revising.

Recent studies have shown that AI writing tools can offer important pedagogical affordances in L2 writing, including feedback support [5], language scaffolding [6], and enhanced learner engagement [7]. At the same time, however, their use has raised substantial concerns related to overreliance [8], academic integrity [9,10], authorship [11], and data privacy [12]. These concerns are particularly salient in educational settings, where students may simultaneously perceive AI tools as highly useful and potentially problematic. As a result, students’ adoption of AI writing tools cannot be understood solely in terms of technological convenience or instructional benefit.

Although prior research has increasingly examined the educational use of GenAI, much of the existing literature has focused either on the pedagogical potential of AI writing tools [1,2,7] or on ethical concerns surrounding their use [8,10,13]. Comparatively less attention has been paid to how students negotiate these two dimensions at the same time when deciding whether and how to engage with AI writing tools in L2 writing-related tasks [14]. This tension points to a central but insufficiently examined issue: students may recognize the usefulness of AI writing tools while simultaneously becoming more aware of their risks.

Accordingly, the present study investigates AI writing tool adoption in Chinese university L2 writing contexts through an extended TAM. By incorporating AI self-efficacy, multidimensional perceived risk, and behavioral engagement, the study seeks to explain not only students’ attitudes toward AI writing tools, but also how their engagement with such tools is associated with self-reported writing-related outcomes.

### 1.2. Research gaps and necessity

Despite the growing body of research on AI in education, at least three important gaps remain in the literature on AI writing tool adoption.

First, existing studies on AI-assisted writing have mainly emphasized instructional affordances [1,2], learner perceptions [7,15], or ethical concerns in isolation [8,10,13]. Fewer studies have examined how perceived usefulness and overall perceived risk jointly shape students’ adoption of AI writing tools [14,16], particularly in L2 writing contexts where efficiency gains and linguistic dependence may coexist [17].

Second, although the TAM has been widely used to explain educational technology adoption [18,19], its application to AI writing tools remains underdeveloped [20,21]. AI writing tools differ from many conventional learning technologies because they are generative, interactive, and capable of co-producing text [22]. These characteristics make students’ perceptions of risk—such as concerns about academic integrity [9,13,23], overreliance [8], and privacy [24]—more central than in many earlier TAM-based studies.

Third, prior research has paid limited attention to what happens after initial acceptance. In particular, the relationship between students’ engagement with AI writing tools and their writing-related outcomes remains insufficiently conceptualized. Existing studies often focus on intention to use or general perceptions [25,26], while providing less integrated explanation of how acceptance may translate into actual behavioral engagement and subsequent positive or negative writing-related experiences.

These gaps indicate the need for a more comprehensive framework that can explain AI writing tool adoption beyond conventional usefulness-based models. Such a framework is especially necessary in higher education, where institutions are increasingly expected to support innovation while also safeguarding academic integrity and responsible AI use.

### 1.3. Research objectives and contributions

This study aims to develop and test an extended TAM-based framework for understanding university students’ adoption of AI writing tools in L2 writing contexts. Specifically, it seeks to examine: (1) how AI self-efficacy shapes perceived ease of use and perceived usefulness [27–29]; (2) how perceived ease of use and perceived usefulness relate to multidimensional perceived risk [8,14,23,24]; (3) whether overall perceived risk influences students’ attitudes toward AI writing tools [11,24,30]; and (4) how attitudes translate into behavioral engagement, which is further associated with self-reported positive and negative writing-related outcomes [21,23–25].

The study makes three main contributions. First, it extends TAM by incorporating AI self-efficacy, multidimensional perceived risk, and behavioral engagement into a single acceptance–use–outcome framework. This extension is designed to capture not only students’ initial acceptance of AI writing tools, but also their subsequent self-reported engagement and perceived writing-related experiences. Second, the study develops a dual-awareness perspective on the usefulness–risk paradox in AI-assisted L2 writing. It argues that perceived usefulness and perceived risk should not be treated only as opposite forces; in generative AI writing contexts, students may recognize the functional value of AI writing tools while also becoming more alert to their academic, ethical, and privacy-related implications. Third, it contributes empirical evidence from Chinese university L2 writing contexts, where the educational use of AI writing tools is expanding rapidly but remains pedagogically and ethically contested [31–33].

This study aims to address the following research questions:

RQ1: How do AI self-efficacy and technology perceptions relate to students’ behavioral engagement with AI writing tools in higher education settings?

RQ2: How is perceived usefulness associated with overall perceived risk in AI writing tool adoption?

RQ3: How is behavioral engagement with AI writing tools associated with students’ perceived writing-related gains and concerns?

## 2. Literature review and hypothesis development

### 2.1. AI Writing tools in L2 writing

AI writing tools have become increasingly visible in L2 writing instruction and practice [22]. Their role extends across multiple stages of the writing process, including idea generation [1], language correction [2], revision [7], and the restructuring of written content [17]. These affordances are particularly relevant in L2 writing, where learners may encounter linguistic limitations, rhetorical difficulties, and reduced confidence during planning, drafting, and revision. Existing studies have reported that AI-supported writing can contribute to grammatical accuracy [15], lexical expression [5], and revision efficiency [22], while also providing immediate feedback and opportunities for individualized support [4].

However, the educational value of AI writing tools cannot be understood solely through their functional affordances. Unlike conventional digital tools that primarily provide resources or predefined feedback, generative AI can actively participate in text production by generating language output, supporting revision, and contributing to the development of written content [21,34]. This more active role in the writing process creates a complex learning context because AI-generated assistance may shape how learners formulate ideas, evaluate suggestions, and negotiate responsibility for the final text [10,13,24]. The same capabilities that help learners address language-related barriers may therefore also raise concerns about authorship, dependence, and appropriate use [8,9,13,24].

Accordingly, recent scholarship has raised concerns about excessive reliance on AI support [8], the need to preserve learners’ critical agency in AI-mediated L2 writing [13], and problematic patterns of AI dependency [24]. Academic integrity has also become a central concern, particularly where AI-generated content may blur the boundary between legitimate assistance and inappropriate authorship practices [3,9]. In addition, privacy and data-related concerns may arise when students enter personally generated texts, assignment drafts, or other potentially sensitive materials into AI systems [35].

Current research indicates that AI writing tools in L2 writing involve both pedagogical affordances and educational concerns. Their generative capabilities may provide linguistic support, feedback, and revision assistance, while also raising concerns about overreliance, academic integrity, authorship, and data privacy [8,10,13,14,24,35]. Thus, students’ evaluations of AI writing tools may extend beyond their perceived usefulness and include consideration of possible consequences associated with their use.

This issue is particularly relevant in Chinese university L2 writing contexts, where AI writing tools are increasingly used for writing, revision, translation, and language polishing, but their pedagogical and ethical boundaries remain under discussion [31,33]. Examining adoption in this context therefore requires attention to both students’ perceived support from AI writing tools and their evaluations of potential use-related concerns.

### 2.2. Theoretical framework

This study is grounded primarily in the TAM [36], which has been widely used to explain users’ acceptance of educational technologies [37–39]. However, AI writing tools differ from conventional digital tools because they not only support learning processes but also generate language output [34], participate in text production [21], and raise concerns related to authorship [10], dependence [13], and privacy [14]. To better capture these characteristics, the present study extends TAM by incorporating AI self-efficacy, multidimensional perceived risk, and behavioral engagement, thereby constructing a more context-sensitive framework for AI writing tool adoption in L2 writing.

#### 2.2.1. Technology acceptance model (TAM).

The TAM [36,40] posits that perceived ease of use (PEOU) and perceived usefulness (PU) are central determinants of users’ attitudes toward a technology and, subsequently, their willingness to engage with it. PEOU refers to the extent to which individuals believe that using a system requires little effort [36], whereas PU refers to the extent to which they believe that the system enhances task performance [40]. TAM has been extensively applied in educational technology research and remains a useful framework for examining students’ acceptance of digital learning tools [38,39,41,42].

However, AI writing tools introduce complexities that are less prominent in earlier TAM applications [34,43]. Unlike conventional instructional technologies, they are generative and semi-autonomous, meaning that students may evaluate them not only in terms of convenience and utility, but also in relation to trust, responsibility, and potential misuse. For this reason, TAM provides a valuable starting point, but requires contextual extension when applied to AI writing tool adoption [44].

#### 2.2.2. Self-efficacy theory.

Self-efficacy refers to individuals’ beliefs about their capability to perform specific tasks successfully [45,46]. In the context of educational technology, self-efficacy has been shown to influence how users’ approach, evaluate, and persist in technology-related activities [47–49]. In AI-mediated learning environments, AI self-efficacy can be understood as students’ confidence in their ability to use AI tools effectively for learning-related purposes.

In the case of AI writing tools, students with stronger AI self-efficacy are more likely to perceive such tools as easier to use and more useful [50], because they feel more capable of generating prompts, interpreting outputs, and integrating AI support into writing-related tasks [32,33]. AI self-efficacy is therefore introduced in this study as an important antecedent to both PEOU and PU.

#### 2.2.3. Perceived risk in AI writing contexts.

Perceived risk refers to users’ expectations of potential negative consequences associated with technology use [9,23,24,30]. In AI writing contexts, such risk is not limited to technical uncertainty, but also involves educational, ethical, and data-related concerns. Students may worry that AI-generated content could weaken independent thinking, create inappropriate dependence [10,51], or blur the boundaries of authorship and academic responsibility [11,13,23,50]. They may also be concerned about the privacy and security of the text they input into AI systems [8,12,30].

Given the multifaceted nature of these concerns, perceived risk in the present study is conceptualized as a multidimensional construct that includes performance-related, academic integrity-related, and privacy-related dimensions. Introducing perceived risk into the model helps explain why students’ responses to AI writing tools may not follow a purely utility-driven logic.

#### 2.2.4. The usefulness–risk paradox in AI-assisted L2 writing.

The usefulness–risk paradox in the present study refers to a dual evaluative condition in which students perceive AI writing tools as instrumentally valuable while simultaneously remaining aware of their potential academic, ethical, and privacy-related risks. This paradox is particularly relevant to AI-assisted L2 writing because generative AI tools can contribute to idea generation, language formulation, revision, and text production rather than merely providing static learning resources or predefined feedback [1,2,7,17,21,34]. The same capabilities that make AI writing tools useful for reducing language-related barriers and supporting writing processes may also create concerns about excessive reliance, authorship ambiguity, inappropriate use, and data-related risks [8–10,13,14,24,35].

In conventional technology acceptance research, perceived usefulness is generally treated as a positive driver of technology acceptance, whereas perceived risk is commonly regarded as a barrier that may reduce favorable evaluations or use intentions [36,40]. This benefit–risk opposition is useful for explaining many conventional technologies, but it is less sufficient for AI writing tools. In generative AI-supported writing, functional value and risk awareness may arise from the same underlying features of the technology. For example, the capacity to generate fluent text and provide immediate writing support may enhance perceived usefulness, while simultaneously making concerns about authorship, academic responsibility, originality, and learner dependence more salient [8–10,13,24].

Accordingly, the paradox does not imply that usefulness and risk are mutually cancelling forces, nor does it assume that students who recognize the usefulness of AI writing tools are unaware of their possible drawbacks. Instead, it captures the possibility that students may hold both evaluations at the same time. As learners become more familiar with the practical value of AI writing tools in drafting, revising, translating, or language polishing, they may also become more attentive to the consequences of relying on AI-generated assistance [10,13,14,24]. This dual-awareness perspective is consistent with recent discussions suggesting that the pedagogical value and potential risks of AI-supported learning may coexist [14,52].

On this basis, the present study treats the relationship between perceived usefulness and overall perceived risk as context-sensitive rather than assuming a uniformly negative association. The purpose is not to claim that usefulness causes risk awareness, but to examine whether students’ perceived functional value and perceived risks are associated in an AI-assisted L2 writing context. This framing extends a benefit-centered application of TAM by recognizing that adoption of generative AI writing tools may involve simultaneous evaluations of support and concern.

#### 2.2.5. Behavioral engagement and perceived writing-related outcomes.

Behavioral engagement in the present study refers to students’ overall self-reported involvement in using AI writing tools for L2 writing-related tasks. In technology adoption research, behavioral intention represents an individual’s willingness to perform or continue a behavior, whereas use behavior refers to the behavioral enactment of technology use [53,54]. These two constructs are theoretically related but conceptually distinguishable because an intention to use a technology does not necessarily correspond to subsequent use under all conditions [53,54].

In the present study, all participants had prior experience using AI writing tools for English writing, revision, translation, or language polishing. Accordingly, behavioral intention is interpreted as students’ intention to continue or further integrate AI writing tools into future L2 writing-related tasks, whereas self-reported actual use refers to their reported current or recent involvement in such tasks. In an ongoing-use context, these two dimensions may share variance because both reflect students’ sustained orientation toward the adoption of AI writing tools, while still representing different temporal aspects of that orientation [53,54].

The present study therefore conceptualizes behavioral engagement as a higher-order self-reported construct comprising behavioral intention and actual use. This specification does not treat behavioral intention and actual use as identical constructs. Rather, it retains them as distinguishable first-order dimensions while modeling their shared variance as an overarching indicator of ongoing adoption engagement. Such a higher-order specification is methodologically appropriate when theoretically related dimensions are empirically difficult to distinguish and when their common variance represents a broader construct.

This modeling decision is also bounded by the cross-sectional design of the study. Because behavioral intention and self-reported actual use were measured at the same time point, the present data cannot establish a temporal sequence in which intention precedes subsequent use. Behavioral engagement should therefore be interpreted as a self-reported indicator of students’ ongoing adoption orientation rather than as objective usage intensity or direct evidence of an intention–behavior causal process.

The writing-related outcomes examined in this study are also self-reported experiences rather than objective writing scores or independently assessed text quality. Perceived positive outcomes include writing efficiency [2,44], confidence during writing [4,55,56], and linguistic support [1,57]. Perceived negative outcomes include overreliance on AI support [8,51,58], reduced originality [29], and concerns about inappropriate use of AI-generated content [59]. Considering both positive and negative outcomes enables a more balanced examination of how students’ ongoing engagement with AI writing tools is associated with their perceived writing-related experiences [8,29,51,58,59].

### 2.3. Hypothesis development

#### 2.3.1. AI self-efficacy, perceived ease of use, and perceived usefulness.

AI self-efficacy reflects students’ confidence in their ability to use AI tools effectively for learning-related purposes. In educational technology research, self-efficacy has consistently been identified as an important antecedent of technology acceptance because users who feel more capable of operating a system tend to perceive it as less difficult and more beneficial. In the context of AI writing tools, students with higher AI self-efficacy are more likely to understand how to formulate prompts, interpret generated responses, and incorporate AI support into their writing practices [34,43]. As a result, they are more likely to perceive such tools as both easier to use and more useful.

Accordingly, the following hypotheses are proposed:

H1: AI self-efficacy is positively associated with perceived ease of use of AI writing tools.

H2: AI self-efficacy is positively associated with perceived usefulness of AI writing tools.

#### 2.3.2. Perceived ease of use and perceived usefulness.

The positive effect of PEOU on PU is a core TAM hypothesis [36,40], widely validated in various domains [37–39]. In AI writing tool contexts, PEOU has been identified as a key predictor of continued use and perceived usefulness [7,15,20,60].

H3: Perceived ease of use is positively associated with perceived usefulness of AI writing tools.

#### 2.3.3. Perceived ease of use, perceived usefulness, and overall perceived risk.

Perceived risk is an important but often underexamined factor in AI writing tool adoption. In general, when a technology is easy to use, users may experience less uncertainty in operating it, which can reduce concerns related to performance and usability [11,23,24,30]. In AI writing contexts, students who perceive AI writing tools as easy to operate may feel more capable of managing prompts, evaluating outputs, and integrating AI assistance into their writing processes. Therefore, perceived ease of use is expected to be negatively associated with overall perceived risk.

Building on the dual-awareness perspective developed in Section 2.2.4, the relationship between perceived usefulness and perceived risk may be context-sensitive in AI writing contexts [14,16]. Rather than assuming that perceived usefulness uniformly reduces risk perceptions, the present study examines whether students who perceive AI writing tools as more useful also report different levels of overall perceived risk. Accordingly, the association between perceived usefulness and overall perceived risk is specified as non-directional. Although the PU-OPR relation is represented as a regression path for model estimation, it is interpreted as an association rather than evidence of a causal ordering.

Accordingly, the following hypotheses are proposed:

H4: Perceived ease of use is negatively associated with overall perceived risk of AI writing tools.

H5: Perceived usefulness is significantly associated with overall perceived risk of AI writing tools.

#### 2.3.4. Overall perceived risk and attitude.

Attitude toward a technology reflects an individual’s overall evaluative orientation toward using it. In technology adoption research, perceived risk is often assumed to negatively influence attitudes [8,13,30] because anticipated negative consequences may undermine users’ trust and willingness to engage [11,23,24]. In AI writing contexts, students who perceive higher levels of performance-related, integrity-related, or privacy-related risk may be less likely to form favorable attitudes toward AI writing tools.

Accordingly, the following hypothesis is proposed:

H6: Overall perceived risk is negatively associated with students’ attitudes toward AI writing tools.

#### 2.3.5. Perceived ease of use, perceived usefulness, and attitude.

Building on the established relationships between PEOU and PU, this hypothesis focuses on the influence of both factors on students’ attitudes toward AI writing tools. According to the TAM, a positive perception of ease of use is likely to enhance the perceived usefulness of a system, which, in turn, can shape attitudes toward using the system [40]. In the context of AI writing tools, this suggests that students who find these tools easier to use may not only appreciate their functionality but also develop a more favorable attitude toward them [16].

Accordingly, the following hypotheses are proposed:

H7: PU is positively associated with ATT.

H8: PEOU is positively associated with ATT.

#### 2.3.6. Attitude, behavioral engagement, and perceived writing-related outcomes.

Positive attitudes toward a technology are generally associated with greater willingness to use it in practice [2,15,20,21]. In the present study, behavioral engagement is conceptualized as a higher-order construct that integrates students’ intention to continue using AI writing tools and their self-reported use tendencies in L2 writing-related tasks. This formulation is suitable for an ongoing-use context in which students have already used AI writing tools and may continue to integrate them into drafting, revising, translating, or polishing activities. Students who hold more favorable attitudes toward AI writing tools are therefore expected to report stronger behavioral engagement.

Such engagement may, in turn, be associated with both positive and negative writing-related experiences. On the positive side, more frequent and active engagement with AI writing tools may provide learners with greater linguistic support [61–63], improved efficiency [2,64,65], and stronger confidence during the writing process [4,7,44]. On the negative side, stronger behavioral engagement may also increase the likelihood of overdependence [8,30,66], reduced originality [18,66,67], or inappropriate reliance on AI-generated content [11,13,24]. Examining both types of perceived outcomes is therefore necessary for a balanced understanding of AI writing tool use.

Accordingly, the following hypotheses are proposed:

H9: Attitude toward AI writing tools is positively associated with behavioral engagement.

H10: Behavioral engagement is positively associated with perceived positive writing-related outcomes.

H11: Behavioral engagement is positively associated with perceived negative writing-related outcomes.

### 2.4. Research model

**Fig 1:**
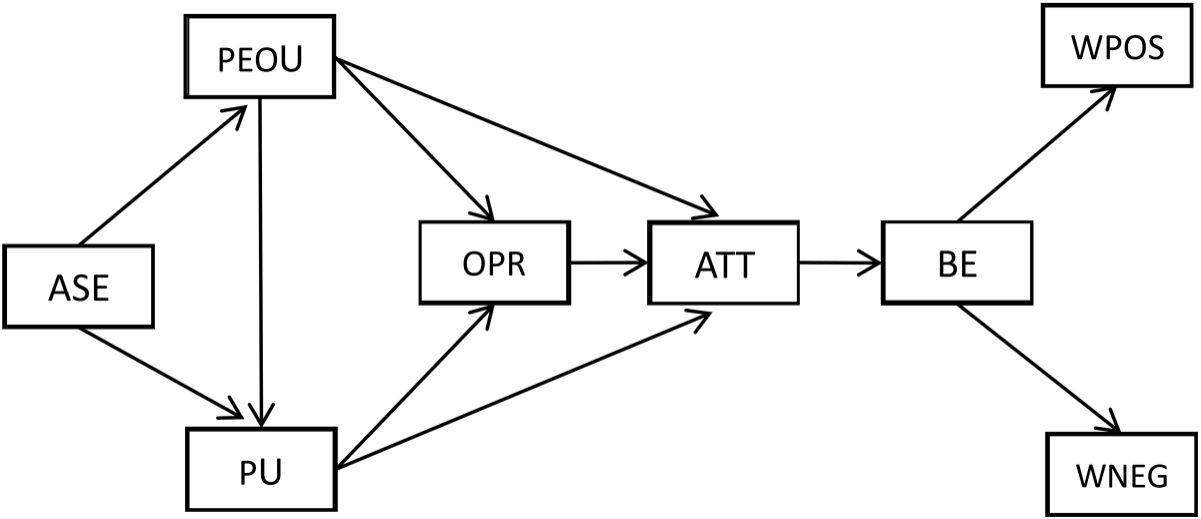
Proposed research model.

Based on the above theoretical considerations and hypotheses, this study proposes an extended TAM-based model in which AI self-efficacy is associated with perceived ease of use and perceived usefulness; perceived ease of use and perceived usefulness are linked to overall perceived risk; overall perceived risk is associated with attitudes; and attitudes are further associated with behavioral engagement. Behavioral engagement is modeled as a higher-order self-reported construct comprising behavioral intention and actual use, and is further associated with both perceived positive and perceived negative writing-related outcomes. The theoretical model is shown in Fig 1. The model includes eight core constructs: AI self-efficacy (ASE), perceived ease of use (PEOU), perceived usefulness (PU), overall perceived risk (OPR), attitude (ATT), behavioral engagement (BE), perceived positive writing-related outcomes (WPOS), and perceived negative writing-related outcomes (WNEG). Arrows represent hypothesized paths.

## 3. Research methodology

### 3.1. Research design and data collection

#### 3.1.1. Sample and sampling procedure.

This study employed a convenience sampling method. The questionnaire was distributed online via Wenjuanxing from January 25 to January 30, 2026, and the survey link was shared through WeChat groups and course instructors across multiple universities in China. The sample comprised undergraduate students from a variety of institution types, including normal (teacher education) universities, comprehensive universities, universities of science and technology, medical universities, finance and economics universities, and arts and sports institutions. The geographical distribution covered several provinces. While convenience sampling limits strict representativeness, this approach is deemed appropriate given the study’s primary aim of testing theoretical relationships within an extended TAM framework rather than estimating population parameters [68]. To be eligible for inclusion, respondents had to report prior experience using AI writing tools (e.g., ChatGPT and Grammarly) for L2 writing-related tasks such as drafting, revising, translating, or language polishing. Responses showing patterned answering, extremely short completion times, or no prior AI writing tool use were excluded from the final analysis. A total of 537 responses were received; after data cleaning, 518 valid responses were retained. Given the cross-sectional and self-report nature of the data, the present study was designed to examine associations among students’ perceptions, attitudes, self-reported engagement, and perceived writing-related outcomes rather than to establish causal effects or objective changes in writing performance.

#### 3.1.2. Data collection process and ethical considerations.

Data were collected using Wenjuanxing, a widely used Chinese online survey platform that supports questionnaire design, online distribution, and response collection. Participants accessed the questionnaire through the previously described online recruitment procedure and completed it voluntarily using their personal devices.

Ethical approval was obtained from the Qilu Normal University Research Ethics Review Committee (Approval No. xsllsc2026-029). Before accessing the survey, participants were presented with an electronic informed consent form on the Wenjuanxing platform. The form explained the purpose of the study, the voluntary nature of participation, the confidentiality of responses, and participants’ right to discontinue participation without penalty. Only participants who selected “Agree” could proceed to the questionnaire; those who selected “Disagree” exited the survey without submitting any responses. The full informed consent materials are provided in S1 Appendix.

### 3.2. Variable measurement

All constructs in this study were measured using established scales, with appropriate adaptations for the context of AI writing tools. All items were measured using a 7-point Likert scale (1 = “Strongly Disagree”, 7 = “Strongly Agree”). A complete list of items and their sources is provided in S1 Appendix.

#### 3.2.1. Construct definitions and measurement tools.

All constructs in this study were measured using multi-item scales adapted from established literature. To ensure contextual appropriateness, the wording of several items was modified to reflect the use of AI writing tools in L2 writing-related tasks. All items were rated on a 7-point Likert scale ranging from 1 (“strongly disagree”) to 7 (“strongly agree”).

AI Self-Efficacy (ASE) refers to an individual’s belief in their ability to successfully use AI writing tools to complete tasks. This scale is adapted from the self-efficacy frameworks of Compeau and Higgins [48] and Bandura [46], containing four items to measure students’ confidence in their ability to use AI tools.

Perceived Ease of Use (PEOU) refers to the degree to which an individual believes that using an AI writing tool is easy and requires little effort. This scale is adapted from the original TAM scale proposed by Davis [40], with four items measuring students’ perception of the ease of using the tool.

Perceived Usefulness (PU) refers to the degree to which an individual believes that using an AI writing tool enhances writing efficiency and quality. This scale is adapted from Davis [40] and Venkatesh [54] and includes four items to measure students’ perceptions of the tool’s usefulness in improving writing outcomes.

Overall Perceived Risk (OPR) was conceptualized as a second-order construct consisting of three first-order dimensions: Performance/Quality Risk (PRQ), Academic Integrity Risk (PRI), and Privacy/Data Risk (PRP). PRQ refers to the possibility that using AI tools may reduce the quality of students’ writing. This dimension was adapted from Featherman and Pavlou [51]. PRI refers to the possibility that using AI tools may lead to academic dishonesty. This dimension was adapted from Holden, Norris [3]. PRP refers to the possibility that using AI tools may expose students to privacy or data-related security concerns. This dimension was adapted from Malhotra [12].

Attitude (ATT) refers to an individual’s overall evaluation of using AI writing tools. Attitude was measured using four semantic differential items adapted from Davis [40] to capture students’ positive or negative evaluations of AI writing tools.

Behavioral Intention (BI) refers to students’ intention to continue using or further integrate AI writing tools into future L2 writing-related tasks and was measured using four items adapted from the UTAUT scale proposed by Venkatesh [54]. Self-reported actual use (AU), in the present study, refers to students’ self-reported frequency of use and depth of interaction with AI writing tools over a specified period and was measured using five items adapted from the UTAUT2 scale proposed by Venkatesh [69]. Because all participants had prior experience using AI writing tools, BI was interpreted as continued or future-oriented use intention rather than initial pre-adoption intention, while AU was interpreted as self-reported current or recent use tendency rather than objective platform-recorded behavior. Given this ongoing-use context, BI and AU were modeled as two first-order dimensions of a higher-order construct, Behavioral Engagement (BE). This specification preserves the conceptual distinction between behavioral intention and actual use while treating them as complementary manifestations of students’ overall behavioral involvement with AI writing tools. Accordingly, BE should be interpreted as self-reported behavioral engagement rather than objective usage intensity.

Perceived Positive Writing-related Outcomes (WPOS) refers to the positive effects perceived by individuals from using AI writing tools. This scale is adapted from Hayes [6], Graham and Perin [70], and Kohnke, Moorhouse [71], containing five items to measure students’ perceptions of the positive impacts of AI tools on their writing.

Perceived Negative Writing-related Outcomes (WNEG) refers to the negative effects perceived by individuals from using AI writing tools. This scale is adapted from Abbas, Jam [8], containing four items to measure students’ concerns about possible negative impacts such as over-reliance or procrastination.

#### 3.2.2. Scale adaptation and pretest.

Because the measurement scales were adapted from previous studies conducted in different technological or educational contexts, several items were revised to fit the specific context of AI writing tool use in L2 writing-related tasks. The initial questionnaire draft was reviewed for clarity and contextual relevance, and minor wording adjustments were made to improve comprehensibility.

A pilot test was then conducted with 51 students who had experience using AI writing tools. Based on their feedback, several items were refined to enhance wording clarity and reduce ambiguity. The final version of the questionnaire was used for formal data collection.

### 3.3. Data analysis procedures

#### 3.3.1. Analysis tools.

IBM SPSS Statistics 27 was used for data cleaning, descriptive statistics, and reliability analysis (Cronbach’s α). Mplus 8.3 was employed for confirmatory factor analysis (CFA) and structural equation modeling (SEM) estimation.

Given that all variables in this study were measured using a 7-point Likert scale, the data are considered ordinal categorical variables rather than continuous variables. Traditional Maximum Likelihood (ML) or Robust Maximum Likelihood (MLR) estimations, commonly used for continuous data, assume multivariate normality and may lead to biased standard errors and inflated chi-square statistics when applied to categorical data [72].

To address this, the Weighted Least Squares Mean and Variance Adjusted (WLSMV) estimation method was used. WLSMV is particularly suitable for analyzing ordinal categorical data and offers several advantages.

First, WLSMV does not assume multivariate normality and estimates parameters using the polychoric correlation matrix, which more accurately reflects the associations between ordinal variables [73]. Second, when response categories are limited (e.g., 5- or 7-point Likert scales) and distributions are skewed, WLSMV provides more accurate parameter estimates and model fit statistics than ML-based methods [74]. Third, WLSMV adjusts chi-square statistics and standard errors, thereby reducing bias caused by non-normality and categorical measurement, especially in complex models with multiple ordinal variables [75].

Given these advantages, WLSMV was selected as the primary estimation method to ensure the accuracy of parameter estimates and model fit.

#### 3.3.2. Common method bias test.

Because all variables were self-reported by the same participants, common method bias (CMB) was considered a potential concern. Following recent methodological recommendations, two complementary approaches were used to assess the potential influence of CMB.

First, Harman’s single-factor test was conducted, with an unrotated exploratory factor analysis (EFA) on all measurement items. The results showed that the first factor explained 30.68% of the total variance, which is below the commonly accepted threshold of 40% [76] and the stricter 50% standard [77]. Although widely used, Harman’s test has been criticized for its low sensitivity in detecting CMB [78], leading us to adopt a more rigorous approach.

Second, the unmeasured latent method factor (ULMC) approach was employed, following the recommendation of Williams and McGonagle [79]. A baseline confirmatory factor analysis (CFA) model was compared with a model including an additional latent method factor loading onto all observed indicators. The baseline model demonstrated good fit: CFI = 0.973, TLI = 0.971, RMSEA = 0.052 (90% CI [0.050, 0.055]), SRMR = 0.045. The ULMC model also showed excellent fit: CFI = 0.979, TLI = 0.977, RMSEA = 0.047 (90% CI [0.044, 0.050]), SRMR = 0.035.

The chi-square difference test was significant (Δχ^2^ = 16435.525, Δdf = 82, p < 0.001), which is expected given the large sample size (N = 518). More importantly, the changes in practical fit indices were minimal: ΔCFI = 0.006, ΔRMSEA = −0.005, and ΔSRMR = −0.010. Following Cheung and Rensvold [80]’s criterion (ΔCFI ≤ 0.01), these results suggest that common method bias does not pose a serious threat.

In addition to these statistical tests, several design features further mitigate concerns about method bias. As noted by Podsakoff et al. [81,82], when complex theoretical relationships are empirically supported, the threat of common method bias is substantially reduced because such patterns are unlikely to be artifactual. In the present study, the complex second-order factor structure (OPR and BE) is unlikely to be explained by a single method factor [83]; and the theoretically meaningful pattern of correlations—such as OPR being non-significant with ATT while PEOU and ATT are highly correlated—aligns with substantive expectations rather than method-driven artifacts. Together, these results indicate that common method bias is not a significant issue in this study and does not materially affect the research conclusions.

#### 3.3.3. Measurement model evaluation.

Before evaluating the structural model, the measurement model was assessed. Reliability was examined using Cronbach’s α and Composite Reliability (CR), with values above 0.70 considered acceptable. Convergent validity was evaluated using standardized factor loadings and Average Variance Extracted (AVE), with factor loadings above 0.60 considered acceptable and those above 0.70 considered preferable, while an AVE greater than 0.50 indicating satisfactory convergent validity. Discriminant validity was assessed using the Fornell–Larcker criterion, which requires that the square root of the AVE for each construct exceed its correlations with other constructs. The results of these analyses are reported in Section 4.2.

After confirming the reliability and validity of the measurement model, the specification of the behavioral component was further examined, given the strong association between behavioral intention (BI) and self-reported actual use (AU). In the present study, two alternative model specifications were considered: a sequential model (ATT → BI → AU) and a higher-order factor model in which BI and AU were treated as indicators of Behavioral Engagement (BE).

As shown in Table 1, the sequential model produced a Heywood case, with the standardized path coefficient for BI → AU exceeding 1 (β = 1.009). Heywood cases typically occur when two latent variables are so highly correlated that they become empirically indistinguishable, resulting in inadmissible parameter estimates [84]. This suggests severe multicollinearity and instability in the sequential specification. By contrast, the higher-order BE model converged normally, with all parameter estimates within the acceptable range, and demonstrated slightly better model fit (CFI = 0.951, RMSEA = 0.070) than the sequential model (CFI = 0.949, RMSEA = 0.071).

**Table 1:** Model comparison: Sequential vs. second-order BE.

Model	CFI	RMSEA	SRMR	Note
Sequential (ATT → BI → AU)	0.949	0.071	0.072	Heywood case (BI → AU > 1)
Second‑order BE	0.951	0.070	0.071	All estimates within range

Note: The sequential model was statistically inadmissible due to the Heywood case; the BE model was therefore retained.

The retention of the higher-order BE specification is supported by both methodological and theoretical considerations. Methodologically, previous research has suggested that combining highly correlated constructs into a higher-order factor is appropriate when they are theoretically related and empirically difficult to distinguish [84,85]. Theoretically, this specification is also consistent with Triandis’s [53] conceptualization of behavioral tendency as a continuum rather than a set of fully discrete stages. In addition, recent research has shown that AI-related behavioral intention may emerge from intersecting configurations of factors rather than from a single linear path [86], further supporting a more integrated representation of behavioral involvement. Taken together, these considerations suggest that, in cross-sectional self-report data, BI and AU may be more appropriately understood as two complementary manifestations of an overarching behavioral engagement construct. Accordingly, the higher-order BE specification was retained in the final analysis.

Importantly, the higher-order BE specification should not be interpreted as claiming that BI and AU are conceptually identical. Rather, it is a conservative measurement specification that retains BI and AU as distinguishable first-order dimensions while modeling their shared variance as a broader self-reported behavioral engagement factor. This approach is appropriate for the present cross-sectional design, because the data do not allow a strict temporal interpretation in which intention necessarily precedes subsequent self-reported actual use. It also avoids overinterpreting a statistically inadmissible sequential path as evidence of a causal behavioral process. Therefore, the higher-order BE model was retained not only because it resolved the Heywood case, but also because it better matched the ongoing and iterative nature of AI writing tool use among experienced student users.

#### 3.3.4. Structural model evaluation and fit indices.

After the measurement model was evaluated and the behavioral engagement specification was confirmed, the structural model was tested to assess the study hypotheses. Model fit was evaluated using the following indices: Chi-square/df ratio (χ²/df) less than 3 for good fit, less than 5 for acceptable fit; Comparative Fit Index (CFI) and Tucker-Lewis Index (TLI) greater than 0.90 for acceptable fit, greater than 0.95 for good fit; Root Mean Square Error of Approximation (RMSEA) less than 0.08 for acceptable fit, less than 0.06 for good fit; Standardized Root Mean Square Residual (SRMR) less than 0.08 for acceptable fit. The significance of path coefficients was determined at a significance level of p < 0.05, with all analyses reporting standardized coefficients.

## 4. Results

### 4.1. Descriptive statistics and correlation analysis

A total of 537 responses were received. After excluding invalid samples (e.g., those with patterned responses, extremely short completion times, or never used AI writing tools), 518 valid responses were retained for analysis, yielding a valid response rate of 96.5% (full data in S2 Appendix). The demographic characteristics of the participants are presented in Table 2. The sample consisted of 41.3% male students, 57.3% female students, and 1.4% students who identified as other or preferred not to disclose their gender. In terms of academic year, 28.6% were freshmen, 15.8% sophomores, 45.6% juniors, and 10.0% seniors. Regarding academic major, 33.0% were from STEM disciplines, 46.5% from the humanities and social sciences, and 20.5% from other disciplines. In terms of experience with AI writing tools, 48.8% reported occasional use, 45.4% frequent use, and 5.8% near-constant use of AI writing tools.

**Table 2:** Demographic profile of the participants.

Variable	Categories	Frequency	Percent
Gender	Male	214	41.3%
	Female	297	57.3%
	Other/Won’t Disclose	7	1.4%
Grade Distribution	Freshman	148	28.6%
	Sophomore	82	15.8%
	Junior	236	45.6%
	Senior	52	10.0%
Major Distribution	STEM	171	33.0%
	Humanities and Social Sciences	241	46.5%
	Others	106	20.5%
Experience with AI Writing Tools	Occasionally Used	253	48.8%
	Frequently Used	235	45.4%
	Almost Always Used	30	5.8%

The means, standard deviations, and correlation coefficients of the key variables are shown in Table 3. From the correlation matrix, it can be observed that most of the core variables exhibit significant relationships, which provides preliminary support for the subsequent hypothesis testing. For example, ASE is significantly positively correlated with PEOU (r = 0.792) and PU (r = 0.699); PEOU is highly correlated with PU (r = 0.771), which is consistent with the basic assumptions of the TAM. ATT shows a strong positive correlation with BE (r = 0.804), indicating that the more positive the attitude, the stronger the overall behavioral engagement. Notably, the correlation between OPR and ATT is relatively weak and negative (r = −0.259). This preliminary observation will be further verified in the subsequent structural model analysis.

**Table 3:** Descriptive statistics and correlation matrix of latent variables.

Variable	Mean	Std.Dev.	PEOU	PU	ASE	OPR	ATT	BE	WPOS	WNEG
PEOU	5.465	0.847	1.000							
PU	5.478	0.893	0.771	1.000						
ASE	5.431	0.915	0.792	0.699	1.000					
OPR	–	–	−0.345	−0.203	−0.260	1.000				
ATT	4.967	1.318	0.833	0.745	0.682	−0.259	1.000			
BE	–	–	0.670	0.599	0.548	−0.208	0.804	1.000		
WPOS	5.304	0.989	0.455	0.407	0.372	−0.141	0.546	0.679	1.000	
WNEG	4.969	1.157	0.263	0.235	0.215	−0.082	0.315	0.392	0.091	1.000

Note: Most correlation coefficients were significant at the p < 0.01 level. Non-significant or marginal correlations are reported as indicated. Means and standard deviations are based on the average scores of the items for each first-order latent variable. OPR and BE are second-order constructs and thus are not applicable for such descriptive statistics.

### 4.2. Measurement model testing

Before conducting structural model analysis, the measurement model was first tested. The sample adequacy test showed that the KMO value was 0.935, and the Bartlett’s test of sphericity was significant (p < 0.001), indicating that the data were suitable for factor analysis.

#### 4.2.1. Reliability analysis.

CFA was performed to test the measurement model. The measurement model showed acceptable fit, with CFI = 0.951, TLI = 0.948, RMSEA = 0.070 (90% CI [0.067, 0.072]), and SRMR = 0.071. The CR and Cronbach’s α coefficients for each latent variable are shown in Table 4. Cronbach’s α values for all constructs exceeded the recommended threshold of 0.70, indicating acceptable internal consistency. CR values were also above 0.70, further supporting construct reliability.

**Table 4:** Reliability and convergent validity of the measurement model.

Latent Variable	Item/Dimension	Standardized Factor Loading	Cronbach’s α	CR	AVE
PEOU	PEOU1	0.909	0.872	0.919	0.739
	PEOU2	0.888			
	PEOU3	0.802			
	PEOU4	0.836			
PU	PU1	0.867	0.900	0.927	0.762
	PU2	0.876			
	PU3	0.862			
	PU4	0.886			
ASE	ASE1	0.890	0.893	0.916	0.733
	ASE2	0.822			
	ASE3	0.835			
	ASE4	0.876			
OPR	PRQ	0.888	–	0.945	0.852
	PRI	0.954			
	PRP	0.926			
ATT	ATT1	0.847	0.954	0.903	0.700
	ATT2	0.834			
	ATT3	0.837			
	ATT4	0.829			
BE	BI	0.865	–	0.880	0.785
	AU	0.907			
WPOS	WPOS1	0.902	0.917	0.940	0.758
	WPOS2	0.857			
	WPOS3	0.894			
	WPOS4	0.845			
	WPOS5	0.855			
WNEG	WNEG1	0.954	0.893	0.913	0.726
	WNEG2	0.777			
	WNEG3	0.848			
	WNEG4	0.818			

Note: OPR and BE are second-order factors; their loadings are standardized coefficients on the first-order factors. All loadings are significant at p < 0.001. CR = Composite Reliability, AVE = Average Variance Extracted.

#### 4.2.2. Validity analysis.

Convergent validity was first assessed using standardized factor loadings and AVE. As shown in Table 4, all standardized factor loadings were above 0.70 (p < 0.001) and statistically significant, while all AVE values exceeded the recommended threshold of 0.50. These results indicate satisfactory convergent validity.

Discriminant validity was then examined using the Fornell-Larcker criterion. As shown in Table 5, the square root of the AVE for each construct was greater than its correlations with other constructs, indicating acceptable discriminant validity. Although some correlations were relatively strong, they did not indicate problematic overlap among the constructs. Taken together, these findings support the adequacy of the measurement model.

**Table 5:** Discriminant validity: Square root of AVE and latent variable correlations.

Variable	PEOU	PU	ASE	OPR	ATT	BE	WPOS	WNEG
PEOU	**0.860**							
PU	0.771	**0.873**						
ASE	0.792	0.699	**0.856**					
OPR	−0.345	−0.203	−0.260	**0.923**				
ATT	0.833	0.745	0.682	−0.259	**0.837**			
BE	0.670	0.599	0.548	−0.208	0.804	**0.886**		
WPOS	0.455	0.407	0.372	−0.141	0.546	0.679	**0.871**	
WNEG	0.263	0.235	0.215	−0.082	0.315	0.392	0.091	**0.852**

Note: The bolded diagonal values are the square roots of AVE, and the lower triangle represents the latent variable correlations.

### 4.3. Structural model testing

After establishing the reliability and validity of the measurement model, the proposed research hypotheses were further tested using structural equation modeling.

#### 4.3.1. Model fit indices.

The structural model demonstrated an acceptable fit to the data. Specifically, the fit indices were as follows: CFI = 0.951, TLI = 0.948, RMSEA = 0.070 (90% CI [0.067, 0.072]), SRMR = 0.071. These values fall within commonly accepted thresholds, suggesting that the proposed model was empirically tenable.

#### 4.3.2. Hypothesis testing results.

**Fig 2:**
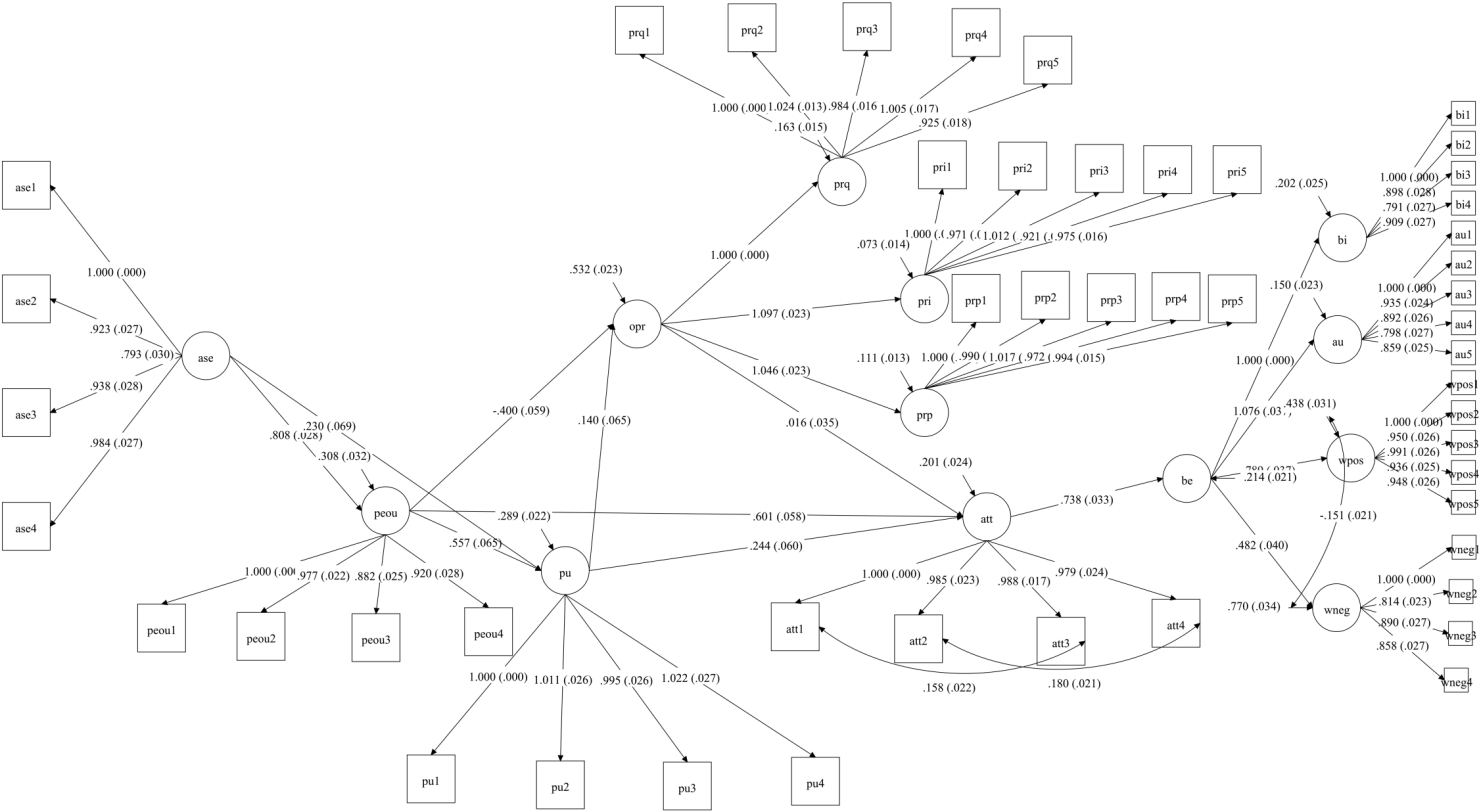
Final structural model with standardized path coefficients.

The standardized path coefficients and hypothesis testing results are presented in Table 6 and Fig 2. All hypotheses were supported except H6.

**Table 6:** Structural model results and hypothesis testing.

Hypothesis	Path Relationship	β	S.E.	t-value	p-value	Result
H1	ASE → PEOU	0.792	0.021	38.270	<0.001	Supported
H2	ASE → PU	0.236	0.071	3.333	0.001	Supported
H3	PEOU → PU	0.584	0.063	9.246	<0.001	Supported
H4	PEOU → OPR	−0.465	0.066	−7.031	<0.001	Supported
H5	PU → OPR	0.155	0.072	2.165	0.030	Supported
H6	OPR → ATT	0.015	0.033	0.451	0.652	Not Supported
H7	PU → ATT	0.250	0.060	4.194	<0.001	Supported
H8	PEOU → ATT	0.645	0.060	10.734	<0.001	Supported
H9	ATT → BE	0.804	0.020	40.636	<0.001	Supported
H10	BE → WPOS	0.679	0.024	28.484	<0.001	Supported
H11	BE → WNEG	0.392	0.030	12.932	<0.001	Supported

Note: ASE = AI self-efficacy, PEOU = Perceived Ease of Use, PU = Perceived Usefulness, OPR = Overall Perceived Risk, ATT = Attitude, BE = Behavioral Engagement, WPOS = Perceived Positive Writing-related Outcomes, WNEG = Perceived Negative Writing-related Outcomes.

Firstly, the path from ASE to PEOU was positive and significant (β = 0.792, p < 0.001), and the path from ASE to PU was also positive and significant (β = 0.236, p = 0.001), supporting H1 and H2. The path from PEOU to PU was positive and significant (β = 0.584, p < 0.001), supporting H3.

Regarding the antecedents of overall perceived risk, the path from PEOU to OPR was negative and significant (β = −0.465, p < 0.001), supporting H4; The path from PU to OPR was also significant and positive (β = 0.155, p = 0.030), supporting H5. This positive association indicates that students who perceived AI writing tools as more useful also tended to report higher awareness of potential risks. Rather than suggesting a simple risk-reduction effect, this finding provides empirical support for the usefulness–risk paradox proposed in this study. Additionally, the path from OPR to ATT was not significant (β = 0.015, p = 0.652), and H6 was not supported.

In the attitude formation section, the paths from PU to ATT (β = 0.250, p < 0.001) and from PEOU to ATT (β = 0.645, p < 0.001) were both positive and significant, supporting H7 and H8.

In the behavioral chain, the path from ATT to BE was positive and significant (β = 0.804, p < 0.001), supporting H9.

Regarding the associations between behavioral engagement and perceived writing-related outcomes, BE was positively associated with both WPOS (β = 0.679, p < 0.001) and WNEG (β = 0.392, p < 0.001), supporting H10 and H11.

Additionally, the R^2^ values for endogenous variables showed good explanatory power, with PEOU (R^2^ = 0.627), PU (R^2^ = 0.615), ATT (R^2^ = 0.720), BE (R^2^ = 0.646), WPOS (R^2^ = 0.461), and WNEG (R^2^ = 0.154). The model explained 12.9% of the variance in OPR (R^2^ = 0.129), suggesting that PEOU and PU accounted for only a limited proportion of students’ overall perceived risk.

## 5. Discussion

Before interpreting the findings, it is important to clarify the scope of inference. Because this study employed a cross-sectional self-report survey design, the structural paths should be interpreted as associations among perceived constructs rather than as evidence of causal effects. In addition, the writing-related outcomes examined in this study refer to students’ perceived positive and negative writing-related experiences, not objective changes in writing proficiency or independently assessed text quality. The discussion below therefore focuses on how students’ perceptions, attitudes, behavioral engagement, and perceived outcomes were related within the proposed extended TAM framework. This study examined university students’ adoption of AI writing tools in L2 writing contexts through an extended TAM framework incorporating ASE, OPR, and BE. Overall, the findings provide partial support for the proposed model and offer a more nuanced account of AI writing tool adoption than conventional usefulness-centered approaches. In particular, the results suggest that students’ responses to AI writing tools are shaped not only by ease of use and functional value, but also by a more complex interplay between utility, risk awareness, and behavioral engagement.

### 5.1. AI self-efficacy as an important antecedent of acceptance

The results showed that ASE is positively associated with both PEOU and PU, supporting H1 and H2. This finding is consistent with prior research suggesting that students who feel more capable of using digital technologies are more likely to approach them with confidence and to perceive them as manageable and beneficial [48,50,87]. In the context of AI writing tools, students with stronger ASE may be better able to formulate prompts, evaluate AI-generated responses, and integrate AI assistance into writing-related tasks. As a result, they are more likely to view such tools as accessible and pedagogically valuable.

This finding also highlights that students’ acceptance of AI writing tools is shaped not only by the features of the technology itself, but also by users’ perceived competence in interacting with it. From a pedagogical perspective, this suggests that strengthening students’ AI literacy and tool-use confidence may be an important condition for more informed and effective adoption.

The relatively high mean score for ASE (M = 5.43) also merits consideration. One possible explanation is that the participants in this study were already relatively familiar with AI writing tools, as all respondents reported prior experience using such tools for L2 writing-related tasks. This pattern is broadly consistent with recent research suggesting that more frequent engagement with generative AI tools is associated with higher confidence in using them [88]. In this sense, the elevated ASE observed in the present sample may reflect students’ accumulated experience with AI-supported writing rather than an unusually high level of AI self-efficacy.

However, this elevated self-efficacy may also reflect a calibration bias common in AI-assisted learning environments. Research on self-assessment accuracy in AI-mediated contexts suggests that while LLM-generated feedback can improve the self-assessment accuracy of lower-performing students, higher-performing students may show decreased accuracy after receiving AI feedback [89], indicating that AI tools may differentially affect students’ calibration of their own abilities. This nuanced finding helps explain why our sample, consisting primarily of regular AI users (94.2% reported at least occasional use), exhibited uniformly high ASE scores—students may be experiencing genuine confidence gains from AI assistance while simultaneously overestimating their unaided capabilities.

Wang and Chuang [43] provide further support for treating AI self-efficacy as a distinct and measurable construct in AI-mediated learning contexts. Students who perceive themselves as competent AI users may also feel more capable of managing potential risks, which may partly help explain the weakened relationship between perceived risk and attitude observed in this study. The significant positive association between PEOU and PU (H3) is consistent with the core proposition of TAM [41]. Students who perceived AI writing tools as easier to use also tended to perceive them as more useful for academic writing. Liu, Liang [16] also found this pathway to be significant in the context of generative AI’s impact on creative writing, reinforcing the connection between ease of use and usefulness in AI tool adoption.

### 5.2. The coexistence of perceived usefulness and risk awareness

The findings regarding OPR are particularly noteworthy. PEOU was negatively associated with overall perceived risk, supporting H4. This suggests that when students perceive AI writing tools as easier to operate, they may experience lower uncertainty and fewer concerns related to usability and performance. In this sample, perceived ease of use was associated with lower overall perceived risk, suggesting that students who found AI writing tools easier to operate tended to report fewer usability-and performance-related concerns.

By contrast, PU was positively associated with OPR, consistent with the non-directional formulation of H5 and revealing a theoretically important pattern. Students who recognized the practical value of AI writing tools also tended to report stronger awareness of potential concerns related to academic integrity, dependence, authorship, and privacy. This result supports the usefulness–risk paradox proposed in this study: greater appreciation of usefulness does not necessarily imply lower concern; in AI-assisted L2 writing, usefulness may coexist with heightened vigilance. This pattern is also compatible with prior research suggesting that greater familiarity with generative AI may heighten users’ awareness of its potential academic and ethical consequences [32]. This pattern also aligns with recent findings by Silva and Sousa [14], who observed similarly complex relationships between usefulness and risk perceptions among Portuguese university students. This interpretation is also broadly consistent with prior research on algorithm aversion, which suggests that people may remain sensitive to the fallibility of algorithmic systems even when such systems perform relatively well overall [90].

This finding adds nuance to conventional TAM-based assumptions, which often imply that usefulness primarily functions as a positive driver of acceptance. In the case of AI writing tools, however, users appear capable of simultaneously perceiving both substantial value and substantial risk. This may reflect the distinctive status of generative AI in education, where the technology is not merely a neutral support tool but also a participant in language production and meaning-making. The present study therefore suggests that AI writing tool adoption should be understood through a dual-awareness lens, in which instrumental value and critical concern develop in parallel.

### 5.3. The non-significant relationship between overall perceived risk and attitude

Contrary to H6, OPR was not significantly associated with students’ attitudes toward AI writing tools. This result suggests that, in this sample, risk awareness did not correspond to less favorable attitudes. One possible explanation is that students may acknowledge concerns about privacy, dependence, and academic integrity while still evaluating AI writing tools positively because of their perceived practical value. This interpretation gains additional support from the relatively high ASE observed in this sample (mean = 5.43 on a 7-point scale). Under such conditions, concerns about privacy, dependence, or academic integrity may be acknowledged cognitively without being strong enough to override the perceived practical value of the tools.

Another possible explanation is that attitude formation in AI writing contexts may be shaped by competing considerations. Students may simultaneously recognize the efficiency and support provided by AI writing tools while also remaining cautious about their misuse. These countervailing evaluations may weaken the direct relationship between overall perceived risk and attitude. This interpretation is consistent with the broader finding of the present study that utility and concern are not necessarily oppositional in students’ evaluations of AI writing tools.

This non-significant relationship should therefore be interpreted carefully. It does not mean that risk is unimportant. Rather, it suggests that perceived risk may operate in a more indirect, conditional, or context-dependent way than originally expected. Future research may therefore benefit from examining whether the influence of risk on attitudes varies across different institutional norms, levels of AI literacy, or patterns of self-reported actual use. Given the cross-sectional and self-reported nature of the data, the present findings should be interpreted cautiously.

Crucially, the non-significance of this path is itself a theoretically meaningful finding. Previous methodological research has suggested that, when complex theoretical relationships are empirically supported, the threat of common method bias is substantially reduced because such patterns are unlikely to be artifactual [82,83]. In the present study, the fact that OPR was associated with PEOU (r = −0.345) and PU (r = 0.155) but was not significantly associated with ATT demonstrates a theoretically meaningful nomological network in which, although common method bias cannot be completely ruled out in self-report survey research, the mixed pattern of significant and non-significant paths, together with the results of the CMB tests, reduces the likelihood that the findings are merely a product of common measurement method. This pattern aligns with recent research in AI ethics literacy [52,91], which suggests that students’ ethical awareness and risk perceptions develop independently from their attitudinal evaluations of AI tools. From a practical perspective, this finding suggests that interventions aimed at reducing perceived risk may not directly improve attitudes; instead, educators should focus on enhancing ASE [50] and critical AI literacy [13] to help students navigate risks while maintaining positive engagement.

### 5.4. Behavioral engagement as an integrated self-reported construct

The use of behavioral engagement as a higher-order construct also requires careful interpretation. In this study, BE integrates behavioral intention and self-reported actual use as two related but distinguishable dimensions of students’ behavioral involvement with AI writing tools. This specification does not imply that intention and use are identical. Instead, it reflects the ongoing-use context of the study, in which all participants had prior experience using AI writing tools for English writing, revision, translation, or language polishing.

In such a context, behavioral intention refers primarily to students’ intention to continue or further integrate AI writing tools into future writing-related tasks, whereas actual use refers to their self-reported current or recent use tendency. AI writing tool adoption is also iterative and task-dependent. Students may move repeatedly between intending to use AI support, consulting AI-generated suggestions, evaluating outputs, and incorporating selected assistance into their writing process. For this reason, a strict temporal sequence from intention to subsequent use is difficult to establish using cross-sectional self-report data.

The higher-order BE specification therefore provides a theoretically and methodologically cautious way to represent students’ overall behavioral involvement. This interpretation is also consistent with the empirical model comparison, where the sequential ATT → BI → AU model produced a Heywood case, while the higher-order BE model converged normally and showed slightly better model fit. At the same time, BE should be interpreted as self-reported behavioral engagement rather than objective usage intensity. Longitudinal data, platform logs, or classroom observation would be needed to examine how intention develops into actual AI writing tool use over time.

### 5.5. The relationship between attitude, behavioral engagement, and perceived writing-related outcomes

In this study, both PU (H7) and PEOU (H8) were positively associated with attitude, supporting the central role of perceived beliefs in the TAM framework [36]. Notably, the association between PEOU and attitude (β = 0.645) was stronger than that between PU and attitude (β = 0.250). In the present sample, this pattern suggests that ease of use may have been relatively more salient than functional value in students’ evaluations of AI writing tools. Attitude was strongly associated with behavioral engagement, supporting H9. Students who reported more favorable attitudes toward AI writing tools also tended to report stronger overall behavioral involvement, including both intention to use and self-reported use tendencies in L2 writing-related tasks.

Behavioral engagement was positively associated with both perceived positive and perceived negative writing-related outcomes, supporting H10 and H11. The positive association between BE and WPOS indicates that students who reported stronger behavioral engagement also tended to report more positive writing-related experiences, such as perceived efficiency, confidence, and linguistic support. However, this finding should not be interpreted as evidence that AI writing tool use objectively improves writing proficiency. The outcome measures capture students’ perceived writing-related gains rather than independently assessed writing performance. For WNEG, the positive association suggests that stronger self-reported engagement was also linked to greater perceived negative writing-related experiences, such as overreliance or reduced originality. This result further supports the need to interpret AI writing tool adoption as a mixed and context-dependent process. This finding is important because it underscores the ambivalent nature of AI writing tool use. Greater engagement was associated not only with perceived benefits, such as writing support, efficiency, and confidence, but also with perceived drawbacks, including overreliance and concerns about inappropriate use. At the same time, the positive association was substantially stronger than the negative one, suggesting that students generally perceived the benefits of engagement as outweighing its disadvantages.

This pattern indicates that behavioral engagement should not be interpreted as uniformly beneficial or uniformly harmful. Consistent with Liu and Liang [16], the present findings suggest that students’ engagement with AI writing tools was associated with a mixed but asymmetrical pattern of perceived experiences, with positive outcomes being more salient than negative ones overall. This balanced interpretation is especially important in current debates on AI in education, where discussions are often polarized between celebration and alarm.

### 5.6. Theoretical implications

This study offers several theoretical implications. Taken together, the findings suggest that the present study contributes primarily as a contextual extension of TAM in AI-mediated L2 writing, while also offering limited refinement of the assumed relationship between usefulness and perceived risk.

First, the study extends TAM in a context-sensitive way by showing that AI writing tool adoption in Chinese university L2 writing contexts cannot be explained adequately through benefit-oriented variables alone. The inclusion of AI self-efficacy, multidimensional perceived risk, and behavioral engagement provides a broader framework for understanding how students evaluate, engage with, and perceive the outcomes of AI writing tools.

Second, the study strengthens the theoretical interpretation of the usefulness-risk paradox. The positive association between perceived usefulness and overall perceived risk suggests that usefulness and risk should not always be treated as opposing evaluations. In AI-assisted writing, the same generative features that make AI tools valuable may also make their academic and ethical implications more visible. This dual-awareness perspective moves the study beyond a conventional benefit-centered TAM explanation and provides a more conceptually robust account of AI writing tool adoption.

Third, by distinguishing between perceived positive and perceived negative writing-related outcomes, the study moves beyond simple intention-based models and broadens the outcome dimension of technology acceptance research. The findings show that students’ behavioral engagement with AI writing tools is associated with both benefits and drawbacks, thereby offering a more differentiated understanding of what adoption may mean in educational settings after initial acceptance has occurred.

### 5.7. Practical implications

The findings also have practical implications for higher education. First, universities should not approach AI writing tool adoption solely as a matter of technological access. Because AI self-efficacy was positively associated with both perceived ease of use and perceived usefulness, institutions should provide students with structured support in prompt design, response evaluation, and responsible integration of AI into writing tasks.

Second, the coexistence of usefulness and risk awareness suggests that pedagogical guidance should go beyond either uncritical encouragement or blanket restriction. Students may already recognize both the value and the risks of AI writing tools; what they need is clearer support in using these tools ethically, reflectively, and strategically.

Third, because behavioral engagement was associated with both positive and negative writing-related experiences, instructional design should aim to maximize the perceived benefits of AI-assisted writing while minimizing overdependence. This may include transparent task guidelines, process-oriented assessment, and explicit discussion of authorship, originality, and revision responsibility.

## 6. Limitations and future research

This study has several limitations. First, the cross-sectional design limits causal interpretation. Although the structural model identifies significant associations among the variables, the data do not allow conclusions about temporal ordering or causal effects. Longitudinal or experimental studies are needed to examine how students’ perceptions and engagement with AI writing tools evolve over time.

Second, all core variables were measured through self-report questionnaires. Although common method bias was assessed using both Harman’s single-factor test and the unmeasured latent method factor approach, self-report data remain subject to recall bias, social desirability bias, and individual differences in self-assessment accuracy. Future studies could combine questionnaire data with interviews, classroom observations, platform usage logs, or trace data.

Another limitation concerns the measurement of behavioral engagement. In this study, BE was modeled as a higher-order construct comprising BI and self-reported AU. Although this specification was theoretically justified and empirically more stable than the sequential model, it does not allow the study to distinguish the temporal process through which intention develops into later actual use. Future research should employ longitudinal designs, experience sampling, or platform-based behavioral logs to examine the transition from intention to actual AI writing tool use more directly.

Third, the study focused on perceived writing-related outcomes rather than objective indicators of writing performance. Therefore, the findings should not be interpreted as evidence that AI writing tool engagement directly improves or weakens writing proficiency. Future research could incorporate writing assessments, teacher ratings, or text-based measures to examine whether perceived benefits and drawbacks correspond to actual changes in writing quality.

Fourth, although overall perceived risk was conceptualized as a multidimensional construct, the present study did not examine in depth whether different types of risk exert distinct effects on students’ attitudes and engagement. Future research may further differentiate among performance-related, integrity-related, and privacy-related concerns, and explore whether their influence varies across contexts. Additionally, future studies could examine conditional effects of AI literacy, institutional policies, or writing task types as moderators to further understand how the usefulness-risk paradox varies across contexts.

Finally, behavioral engagement in this study was measured through self-reported use tendencies and practices. Future studies could combine self-report data with behavioral logs, usage traces, or classroom-based observations to provide a more robust picture of actual AI writing tool engagement.

## 7. Conclusion

This study developed and tested an extended TAM-based framework to examine university students’ adoption of AI writing tools in L2 writing contexts. Based on cross-sectional self-report data from Chinese undergraduate students, the findings show that AI self-efficacy was positively associated with perceived ease of use and perceived usefulness, and that students’ attitudes toward AI writing tools were strongly associated with their self-reported behavioral engagement. More importantly, the study found a positive association between perceived usefulness and overall perceived risk. This pattern supports the usefulness–risk paradox proposed in the study, suggesting that students may recognize the practical value of AI writing tools while also remaining aware of their academic, ethical, dependence-related, and privacy-related implications. In addition, behavioral engagement was associated with both perceived positive and perceived negative writing-related outcomes, indicating that AI writing tool adoption may involve both perceived benefits and perceived drawbacks.

These findings contribute to research on AI-assisted writing and technology acceptance by showing that AI writing tool adoption in L2 writing contexts cannot be fully explained through a benefit-centered model. At the same time, the findings should be interpreted within the limits of the study design. Because the study relied on cross-sectional self-report data, it cannot establish causal effects or objective changes in writing performance. Future research should integrate longitudinal designs, experimental evidence, writing assessments, and behavioral log data to further examine how students engage with AI writing tools and how such engagement relates to actual writing development.

## Supporting information

S1 Appendix Questionnaire. (DOCX)

S2 Appendix Data. (XLSX)
