# Supplementary material for: Understanding the usefulness–risk paradox in AI writing tool adoption: An extended technology acceptance model study in Chinese University L2 writing contexts
Source: PLoS One. 2026 Aug 7;21(8):e0355494. doi: 10.1371/journal.pone.0355494 (PMC13450789; doi:10.1371/journal.pone.0355494)
Supplement: Supplementary file 1 [file pone.0355494.s001.docx]

**S1 Appendix. Questionnaire**

**Informed Consent Form**

Dear Participant,

Thank you for considering participation in this research study. Before proceeding with the questionnaire, please carefully read the following information.

**1.Purpose of the Study**

This study investigates university students’ adoption of AI writing tools in English writing-related tasks. It aims to understand how students perceive the usefulness and risks of AI writing tools, and how these perceptions are associated with their attitudes, behavioral engagement, and perceived writing-related outcomes.

**2.Voluntary Participation**
Your participation is entirely voluntary. You may choose not to participate, or you may withdraw from the survey at any time before submitting your responses, without any penalty or negative consequences.

**3.Confidentiality and Anonymity**

This questionnaire is anonymous. No personally identifiable information will be collected. All responses will be kept confidential and used only for academic research purposes.

**4.Data Collection**
This questionnaire is administered via Wenjuanxing. The formal survey period is from January 25 to January 30, 2026.

**5.Data Usage**
The data collected in this study will be used only for academic research, including statistical analysis, scholarly reporting, and supporting the findings of this study.

**6.Consent**
By selecting “Agree” and proceeding to the questionnaire, you confirm that you have read and understood the information above and voluntarily agree to participate in this study.

**After carefully reviewing the Informed Consent Form above, do you voluntarily agree to participate in this study under the stated terms?**

A. Agree

B. Disagree

**Questionnaire on AI Writing Tool Adoption in English Writing Contexts**

This questionnaire consists of two sections. Please select the option that best reflects your situation. For the Likert-scale items, indicate the extent to which you agree or disagree with each statement.

**Section 1**

**Background Information**

1. Gender

A. Male
B. Female
C. Other / Prefer not to disclose

2. Grade
   A. Freshman
   B. Sophomore
   C. Junior
   D. Senior
3. Major
   A. English Major

B. Other Foreign Language Majors (e.g., Japanese, French, Russian, etc.)

C. Humanities and Social Sciences Majors (e.g., Chinese Language and Literature, History, Philosophy, Education, Management, Economics, etc.)

D. Science, Engineering, and Medical Majors (e.g., Science, Engineering, Information, Medicine, Agriculture, etc.)

E. Arts and Sports Majors

F. Other (please specify): __________

4. Self-rated English Writing Proficiency

A. Very weak

B. Weak

C. Fair

D. Good

E. Very good

5. **How often do you use AI writing tools for English writing-related tasks?（Frequent use）**
   A. Never used
   B. Occasionally used
   C. Frequently used

D. Used in almost every writing task

6. Which AI writing tools have you used most often?
   A. Deepseek
   B. Kimi
   C. Tongyi Qianwen / Qwen
   D. iFlytek Spark / Xunfei Xinghuo

E. ERNIE Bot / Wenxin Yiyan

F. Doubao

G. Pigai

H. Grammarly

I. ChatGPT

J. Gemini

K. QuillBot

L. Grok

M. Claude

N. Other (please specify): __________

7. **For which English writing tasks do you mainly use AI writing tools? (Select all that apply)**
   A. In-class English essays
   B. English exam writing (e.g., final exams, CET-4/6, IELTS, TOEFL, etc.)
   C. English course papers / course reports
   D. Reflective journals / writing practice logs
   E. Daily English writing practice (e.g., English emails, social media posts in English, etc.)
   F. Other (please specify): __________
8. **Type of Institution**

**A.** Normal / Teacher Training University

**B.** Comprehensive University

**C.** Science and Engineering University

**D.** Medical University

**E.** University of Finance and Economics

**F.** University of Arts and Sports

G. Other (please specify): __________

**Section 2**

**Scale Items**

**Response Scale:**
1 = Strongly disagree
2 = Disagree
3 = Somewhat disagree
4 = Neutral
5 = Somewhat agree
6 = Agree
7 = Strongly agree

**Perceived Ease of Use (PEOU)**

PEOU1. Learning to operate the AI writing assistant is easy for me.
PEOU2. I find it easy to get the AI writing assistant to do what I want it to do.
PEOU3. My interaction with the AI writing assistant is clear and understandable.
PEOU4. It is easy for me to become skillful at using the AI writing assistant.

**Perceived Usefulness (PU)**

PU1. Using the AI writing assistant improves the efficiency of my English writing.
PU2. Using the AI writing assistant helps me produce better organized writing.
PU3. Using the AI writing assistant enhances the overall quality of my writing tasks.
PU4. Using the AI writing assistant makes my writing tasks easier to accomplish.

**AI Writing Self-Efficacy (ASE)**

ASE1. I am confident in my ability to use the AI writing assistant effectively.
ASE2. I can learn new functions of the AI writing assistant quickly.
ASE3. I can use the AI writing assistant even without others’ help.
ASE4. I feel capable of guiding the AI writing assistant with appropriate prompts.

**Performance / Quality Risk (PRQ)**

PRQ1. The AI writing assistant may generate inaccurate or misleading content.
PRQ2. The AI writing assistant may produce writing that does not match academic standards.
PRQ3. I am concerned that AI-generated writing may reduce the quality of my assignments.
PRQ4. The AI writing assistant may generate content with factual errors.
PRQ5. I need to double-check AI-generated writing to ensure its accuracy.

**Academic Integrity Risk (PRI)**

PRI1. Using the AI writing assistant may cause my work to be questioned for originality.
PRI2. I worry that using AI writing assistance may violate course or institutional rules.
PRI3. I am concerned that AI writing use could lead to academic misconduct accusations.
PRI4. I am unsure about how much AI assistance is acceptable in academic writing.
PRI5. I worry that teachers may penalize AI-assisted writing.

**Privacy / Data Risk (PRP)**

PRP1. I am concerned that my writing data may be collected without my consent.
PRP2. I worry that my personal information could be leaked when using the AI writing assistant.
PRP3. I feel uneasy about submitting my writing content to the AI writing assistant.
PRP4. I worry that my writing data may be stored permanently by AI service providers.
PRP5. I am concerned about who can access my writing content after I submit it to AI tools.

**Attitude (ATT)**

ATT1. Using the AI writing assistant for English writing is a good idea.
ATT2. I like using the AI writing assistant in my English writing tasks.
ATT3. Using the AI writing assistant in my English writing tasks is a pleasant experience.
ATT4. Using the AI writing assistant for English writing is a worthwhile choice.

**Behavioral Intention (BI)**

BI1. I intend to continue using the AI writing assistant in my future English writing tasks.
BI2. I plan to use the AI writing assistant for a wide range of English writing tasks (e.g., drafting, revising, polishing).
BI3. Given the choice, I would prefer to use the AI writing assistant rather than write entirely by myself.
BI4. I would recommend the AI writing assistant to my classmates for English writing.

**Actual Use (AU)**

AU1. I use the AI writing assistant regularly when completing English writing assignments.
AU2. I use the AI writing assistant at multiple stages of my writing process.
AU3. I rely on the AI writing assistant in most writing-related tasks.
AU4. I use AI writing assistance in both drafting and revising stages.
AU5. I use the AI writing assistant for longer writing tasks rather than only short exercises.

**Perceived Positive Writing-Related Outcomes (WPOS)**

WPOS1. Using the AI writing assistant helps me generate clearer ideas for my writing.
WPOS2. Using the AI writing assistant improves my ability to revise my writing.
WPOS3. Using the AI writing assistant helps me better understand how to structure an English essay.
WPOS4. I can apply writing strategies learned from AI assistance when writing independently.
WPOS5. Using the AI writing assistant helps me develop better argumentation in writing.

**Perceived Negative Writing-Related Outcomes (WNEG)**

WNEG1. When working on English writing tasks, the availability of AI-generated content makes me more likely to delay starting the writing process.
WNEG2. I become more easily distracted or interrupted when using an AI writing assistant, which lowers my productivity.
WNEG3. After using AI writing assistants, I find myself less inclined to memorize writing conventions or complex ideas, as I believe I can always search for them later.
WNEG4. I am concerned that prolonged use of AI writing assistants could weaken my mental agility and memory for creative ideas.

**Note.**
All scale items were measured on a 7-point Likert scale ranging from 1 (strongly disagree) to 7 (strongly agree). The questionnaire was administered in Chinese in the formal survey, and the English version is provided here for reporting purposes. Item wording was adapted to fit the context of AI writing tools in English writing-related tasks. The full item pool and sources are consistent with the measurement appendix and the research design reported in the article.
